# Ecological data in support of an analysis of Guinea-Bissau׳s medicinal flora

**DOI:** 10.1016/j.dib.2016.03.077

**Published:** 2016-03-30

**Authors:** Luís Catarino, Philip J. Havik, Bucar Indjai, Maria M. Romeiras

**Affiliations:** a University of Lisbon, Faculty of Sciences, Centre for Ecology, Evolution and Environmental Changes (cE3c), Lisbon, Portugal; b Universidade NOVA de Lisboa, Instituto de Higiene e Medicina Tropical, Centre for Global Health and Tropical Medicine (GHTM), Lisbon, Portugal; c Instituto Nacional de Estudos e Pesquisa, Centro de Estudos Ambientais e Tecnologia Apropriada (INEP/CEATA), Bissau, Guinea-Bissau; d University of Lisbon, Faculty of Sciences, Biosystems and Integrative Sciences Institute (BioISI), Lisbon, Portugal

**Keywords:** West Africa, Ethnobotanical data, Useful plants, Geographical distribution, Vegetation types

## Abstract

This dataset presents an annotated list of medicinal plants used by local communities in Guinea-Bissau (West Africa), in a total of 218 species. Data was gathered by means of herbarium and bibliographic research, as well as fieldwork. Biological and ecological information is provided for each species, including in-country distribution, geographical range, growth form and main vegetation types. The dataset was used to prepare a paper on the medicinal plants of Guinea-Bissau “Medicinal plants of Guinea-Bissau: therapeutic applications, ethnic diversity and knowledge transfer” (Catarino et al., 2016) [1]. The table and figures provide a unique database for Guinea-Bissau in support of ethno-medical and ethno-pharmacological research, and their ecological dimensions.

**Specifications Table**

**Table:** 

Subject area	*Biology*
More specific subject area	*Ethnobotany*
Type of data	*Table, Figures, Images*
How data was acquired	*Fieldwork, herbarium data, bibliographic research*
Data format	*Filtered and organised*
Experimental factors	*Field, herbarium and bibliographic data was filtered and classified.*
Experimental features	*Ethnobotanical and ecological data were collected during fieldwork carried out in different regions of Guinea-Bissau. Also, semi-structured interviews, observations and guided field surveys were conducted with local healers.*
Data source location	*Latitude and longitude of Guinea-Bissau is 10°59′–12°20′N and 13°40′–16°43′W.*
Data accessibility	*Data are included in this article*

**Value of the data**

• This dataset is the first comprehensive record of Guinea-Bissau׳s medicinal flora totalling 218 plant species.
• It includes information on the growth form, vegetation types, in-country distribution, distribution range, plant parts used, and other uses of species besides medicinal.
• Species were recorded for remote and hitherto under-researched areas.
• This dataset can be used as primary source guide for the biological and ecological contexts of ethno-medical research in Guinea-Bissau and West Africa.

## 1 Data

The comprehensive list of plant species used in traditional medicine in Guinea-Bissau is presented in Table 1, focuses on biological and ecological aspects of the original dataset. It identifies medicinal and non-medicinal uses of recorded species, as well as the plant parts used, growth form, main vegetation types and geographical distribution. The dataset was specially prepared for a recent study (see [1]), based upon information gathered during fieldwork performed in the country, from 1990s to the present, by the authors, complemented with data from herbarium vouchers (LISC Herbarium, University of Lisbon) as well as from published sources. Samples were collected in various geo-morphological areas covering a wide range of vegetation types in Guinea Bissau, inhabited by more than thirty ethnic groups.

## 2 Experimental design, materials and methods

### 2.1 Study area

**Fig. 1:**
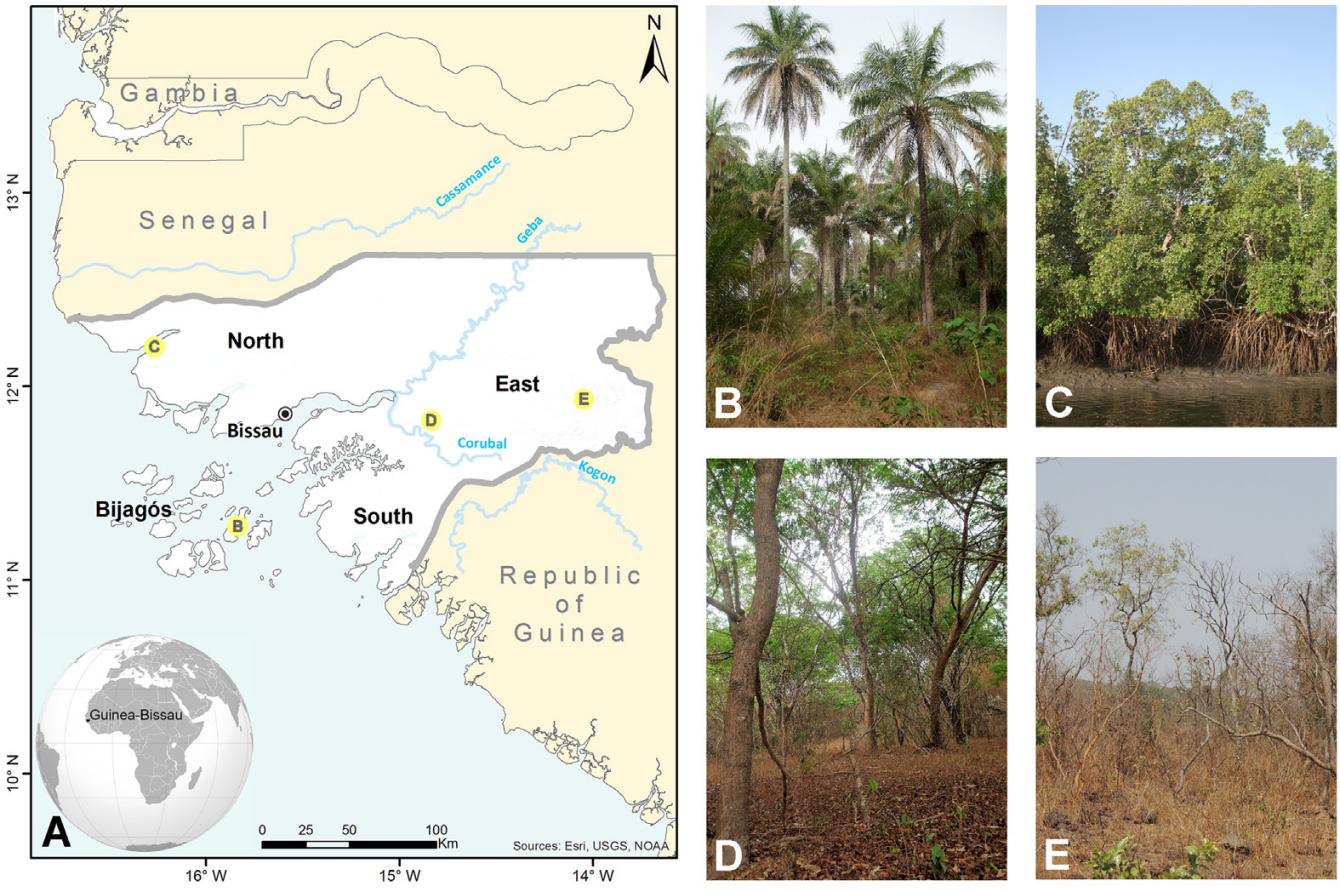
Map of Guinea-Bissau (A – left). Some of the main vegetation types (right): (B) palm groves, Bijagós Islands; (C) mangroves, Cacheu Natural Park; (D) woodlands, Dulombi Park; (E) savanna woodlands, Boé Park (photographs L.Catarino).

Guinea-Bissau is located in the Northern Intertropical Zone of West Africa. It borders on the Republic of Senegal to the North, the Republic of Guinea to the East and South and by the Atlantic Ocean to the West [2]. It covers 36,125 km^2^ and the country encompasses continental mainland (North, South, and Eastern regions) and a group of 40 islands, the Bijagós Archipelago (Fig. 1A).

Four different geo-morphological areas are identified for Guinea Bissau, i.e. the Bijagós Islands covered by palm groves (Fig. 1B) and dry forests surrounded by mangroves; the low-lying, littoral region with extensive mangroves dissected by rivers and creeks (Fig. 1C); a transitional zone extending in an easterly direction covered in woodlands (Fig. 1D); the savanna plains in the East and South-East sloping upwards to a maximum height of 300 m (Fig. 1E).

### 2.2 Guinea-Bissau׳s medicinal flora: vegetation types and species distribution

#### 2.2.1 Flora and vegetation types

Guinea-Bissau׳s vascular flora comprehends 1524 taxa, of which 1391 are native [3]. The main vegetation types occurring in Guinea-Bissau were documented during several field surveys and are identified in Table 1, in which the species are assigned to vegetation types in accordance with the following classes: a) forest; b) woodland; c) savanna woodland; d) wet grass savanna; e) palm grove; f) riparian forest; g) mangrove and h) ruderal habitats (see Fig. 1B–E). In terms of surface area, savanna woodland dominates, followed by woodland, mangrove, herbaceous savanna and dry forest, mainly found in the South [3].

#### 2.2.2 Medicinal plants

**Fig. 2:**
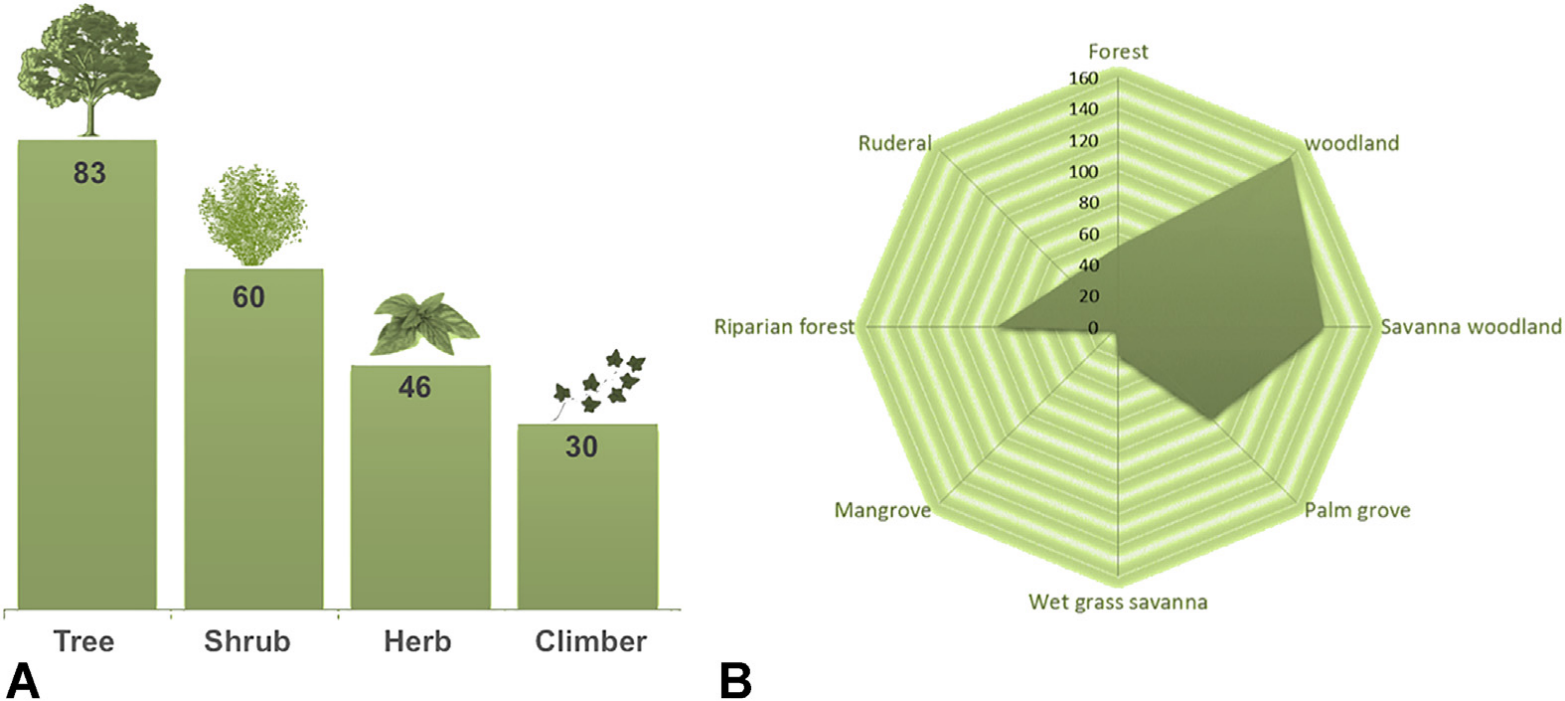
Distribution of medicinal plants in Guinea-Bissau by life-form (A) and vegetation types (B).

**Fig. 3:**
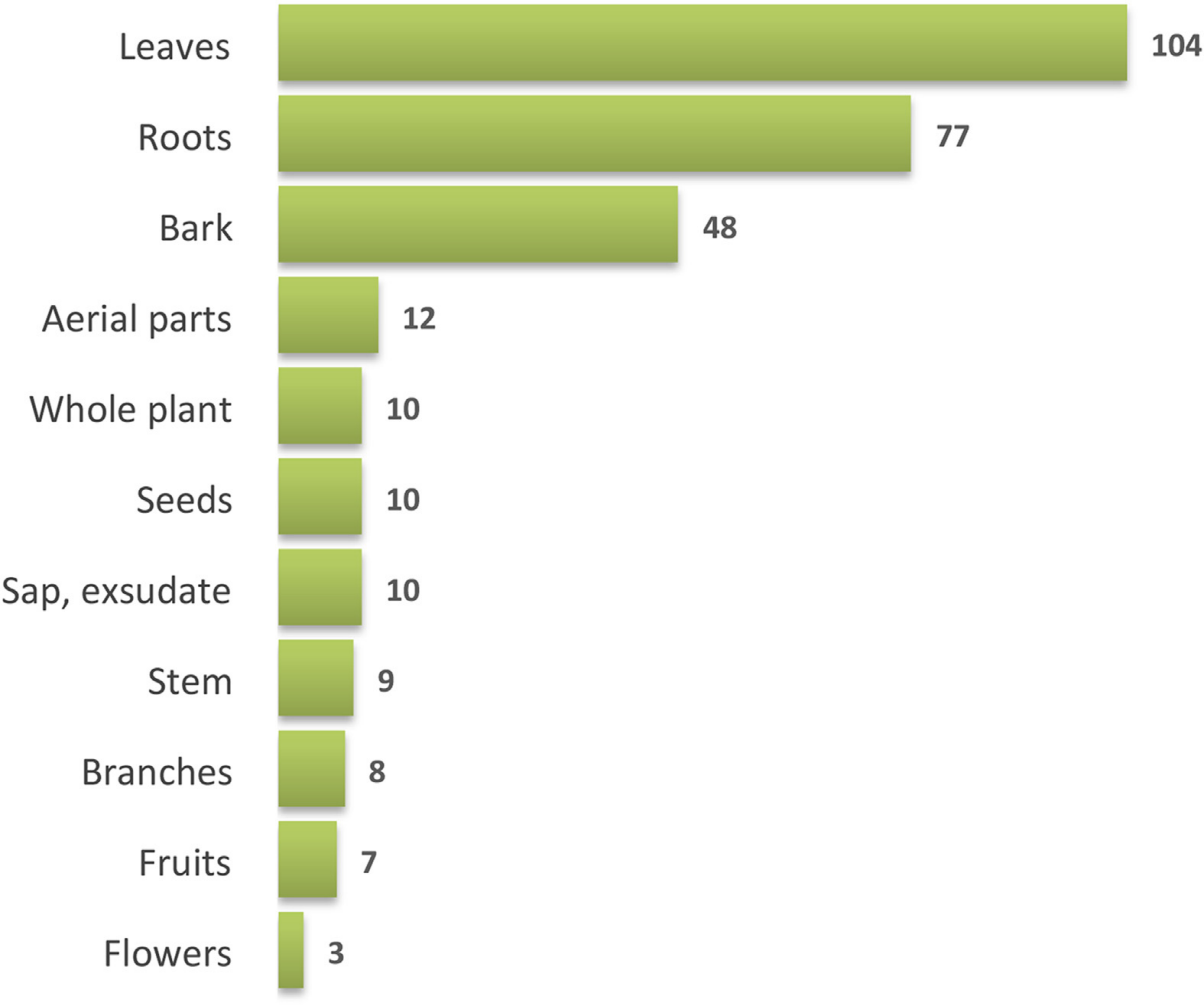
Plant parts used in traditional medicine in Guinea-Bissau.

A total of 218 medicinal plants as used in traditional medicine in Guinea-Bissau were identified in a recent study [1]. Ecological and distributional data are presented in Table 1, and in Fig. 2. Most the medicinal plants are trees (38%; 3 palms are included in this group), followed by shrubs (27%), herbs (21%) and climbers (14%) (Fig. 2A). The main vegetation types in which the medicinal plants species occur are woodland and savanna woodland, but species are also found in areas covered in palm groves, forest and riparian forest. Almost all the species occur in more than one vegetation type and half of them in both woodland and savanna woodland (Fig. 2B). The plant-parts most used are leaves, followed by roots and stem bark. In the case of herbaceous species, usually the whole plant and the aerial parts are used (Fig. 3).

Given that medicinal plants are often used for other than medicinal purposes, these are identified in Table 1: a) Handicrafts – plant parts used for handicrafts and utensils (16 species); b) Food – whole plant or plant part edible (52 species); c) Beverages – plant parts used for beverages without therapeutic purposes (6 species); d) Rituals and beliefs – whole plants or plant part used in traditional ceremonies or for magic-religious purposes (7 species); e) Phytochemical – plants used by their chemical compounds (21 species) and f) Timber – species used for wood (10 species).

#### 2.2.3 Geographical distribution

For each of the medicinal species considered in our study, information was compiled on their distribution in Guinea-Bissau: North, South, and East (continental regions) and the Bijagós Archipelago (Fig. 1A). Moreover the 218 medicinal species were categorised into its distribution range (phytogeography), which allow us to characterise the diversity of the climatic, edaphic and floristic-ecological conditions where they occurs. Therefore, the distribution types used are based on those of White (see [4]) for African phytogeographic regions, namely: a) Afro-American (=Afro-Neotropical) – species distributed in the tropical or subtropical regions of Africa and America; b) Amphi-Atlantic – species occurring in the coastal regions on both sides of the Atlantic Ocean, in Africa and America; c) Afrotropical – species distributed in the African continent; d) Guinean sub-region; e) Guineo-Congolian region; f) Guineo-Congolian and Sudano-Zambezian regions; g) Guinean sub-region and Zambezian region; h) Paleotropical – species occurring in the tropical or subtropical regions of Africa, Asia, Europe and Oceania; i) Pantropical – species occurring in the tropical or subtropical regions all over the world; j) Sudanian and the Guinean sub-region; l) Sudanian and the Guineo-Congolian regions; m) Sahelian and Sudanian regions; and o) Sudanian and Zambezian regions.

### 2.3 Data collection

The study of ecological aspects associated with Guinea-Bissau׳s medicinal flora was carried out by means of in-country fieldwork, the collection and study of herbarium specimens and a detailed inventory of published sources. An initial countrywide study of the country׳s flora was conducted between 1997 and 2007 (for more details see [3]). Recent fieldwork included visits to communities in different areas of the country, interviews with local community members and healers, and observations in loco*.* Close collaboration with IBAP staff allowed for the gathering of information on ecological conditions and conservation measures implemented in protected areas. The data presented in Table 1 was assembled by means of the following sources:

1) *Fieldwork.* Ecological and distribution data was collected during the fieldwork carried out during the last two decades in different regions of Guinea-Bissau. The in-country collection of ethnobotanical data relied on semi-structured interviews, in-field observations and guided field surveys were conducted with local healers and members of local communities.
2) *Herbarium Specimens.* A detailed review was undertaken of plant specimens at the LISC Herbarium (University of Lisbon) that contain the most important worldwide collection from this country. After examining each record in the herbarium׳s Guinea-Bissau collection, a critical and updated review of Guinea-Bissau׳s plants, their growth-environment, their medicinal relevance and alternative uses was implemented.
3) *Bibliographic Review.* A review of publications on medicinal flora of Guinea-Bissau covering the period from 1880s to the present was carried out (for more details see [1]). Moreover, a review of the literature was carried out to investigate previous studies׳ classifications of vegetation types [e.g. [3], [5], [6], [7]], and ecological data collected during fieldwork was complemented with a study of the literature on ecology, biodiversity and the impact of climate change in Guinea Bissau.

Finally all the available data on Guinea-Bissau׳s medicinal flora, including ecological knowledge obtained from specimen vouchers and published literature, as well as the authors׳ field experience, were incorporated into a database (Table 1).

## Figures and Tables

**Table 1:** Ecological data related with the Guinea-Bissau׳s medicinal flora, with references to other uses of species.

**Species**	**Family**	**Growth form**	**Habitat types**	**Distribution Guinea-Bissau**	**Distribution range**	**Plant parts used in traditional medicine**	**Other uses of medicinal plants**	**Herbarium voucher**
*Abelmoschus esculentus* (L.) Moench.	Malvaceae	Herb	Cultivated	North, South, East, Bijagós	Introduced, Native to Asia	Seeds	Food	Moreira 284
*Abrus precatorius* L. subsp*. africanus* Verdc.	Fabaceae	Climber	Woodland, Savanna Woodland	North, South, Bijagós	Pantropical	Aerial parts of plant		Indjai 17
*Acacia macrostachya* Rchb. ex DC.	Fabaceae	Tree	Woodland, Savanna Woodland	North, South, East	Sudanian Region	Roots		Catarino 1269
*Acridocarpus plagiopterus* Guill. & Perr.	Malpighiaceae	Shrub	Forest, Woodland, Savanna Woodland, Palm Grove, Gallery Forest	North, South, East, Bijagós	Guinean Sub-Region	Leaves	Phytochemical	Diniz & Gonçalves 1861
*Acridocarpus smeathmannii* (DC.) Guill. & Perr.	Malpighiaceae	Shrub	Savanna Woodland, Gallery Forest	North	Guineo-Congolian Region	Leaves		Diniz & Gonçalves 1863
*Adansonia digitata* L.	Malvaceae	Tree	Savanna Woodland, Cultivated	North, South, East, Bijagós	Afrotropical	Leaves	Food	Sané 74
*Adenia lobata* (Jacq.) Engl.	Passifloraceae	Climber	Forest, Woodland, Savanna Woodland, Palm Grove, Gallery Forest	North, South, Bijagós	Guineo-Congolian Region	Roots, Sap		Catarino 1664
*Afraegle paniculata* (Schumach. & Thonn.) Engl.	Rutaceae	Tree	Forest, Woodland, Savanna Woodland	North, South, East	Guineo-Congolian Region	Roots		Diniz & Gonçalves 1880
*Aframomum alboviolaceum* (Ridl.) K.Schum.	Zingiberaceae	Herb	Woodland, Savanna Woodland	South	Afrotropical	Roots	Food	Catarino 1385
*Aframomum rostratum* K.Schum.	Zingiberaceae	Herb	Woodland, Savanna Woodland	North, South	Guinean Sub-Region	Roots	Food	Martins et al. 622
*Afzelia africana* Sm. ex Pers.	Fabaceae	Tree	Woodland, Savanna Woodland, Gallery Forest	North, South, East, Bijagós	Sudanian/Guinean Sub-Region	Bark	Timber, Handicrafts	Catarino 495
*Agelaea pentagyna* (Lam.) Baill.	Connaraceae	Shrub	Forest, Gallery Forest	South	Afrotropical	Leaves		Catarino 1684
*Albizia adianthifolia* (Schum.) W.Wight	Fabaceae	Tree	Forest, Woodland	North, South, East, Bijagós	Afrotropical	Leaves		Catarino 1562
*Alchornea cordifolia* (Schumach. & Thonn.) Müll.Arg.	Euphorbiaceae	Shrub	Forest, Woodland, Plam Grove, Gallery Forest	North, South, East, Bijagós	Afrotropical	Stem, Seeds	Phytochemical	Catarino 1549
*Allophylus africanus* P.Beauv.	Sapindaceae	Shrub	Forest, Woodland, Savanna Woodland, Palm Grove, Gallery Forest, Wet Grass Savanna	North, South, East, Bijagós	Afrotropical	Leaves		Indjai 26
*Alstonia boonei* De Wild.	Apocynaceae	Tree	Woodland, Savanna Woodland, Gallery Forest	North, South, East, Bijagós	Afrotropical	Sap	Timber, Handicrafts	Catarino 738
*Alstonia congensis* Engl.	Apocynaceae	Tree	Forest, Gallery Forest	South, East, Bijagós	Guineo-Congolian Region	Bark, Roots	Timber, Handicrafts	Catarino 1513A
*Ampelocissus multistriata* (Baker) Planch.	Vitaceae	Climber	Savanna Woodland	North, South, Bijagós	Sudanian/Zambezian Regions	Leaves		Martins & Moreira 998
*Anacardium occidentale* L.	Anacardiaceae	Tree	Cultivated	North, South, East, Bijagós	Introduced, Native to America	Leaves, Bark	Food, Beverages	Catarino 1922
*Anisophyllea laurina* R.Br. ex Sabine	Anisophylleaceae	Tree	Forest	South, Bijagós	Guinean Sub-Region	Leaves	Food	Catarino 1698
*Annona glabra* L.	Annonaceae	Shrub	Cultivated	North, South, East, Bijagós	Introduced, Native to America	Roots	Food	Moreira 38
*Annona muricata* L.	Annonaceae	Tree	Cultivated	North, South, East, Bijagós	Introduced, Native to America	Leaves	Food	Diniz et al. 2595
*Annona senegalensis* Pers.	Annonaceae	Shrub	Savanna Woodland	North, South, East, Bijagós	Afrotropical	Roots, Leaves, Flowers	Food	Catarino 1352
*Antherotoma senegambiensis* (Guill. & Perr.) Jacq.-Fél.	Melastomataceae	Herb	Woodland, Palm Grove, Wet Grass Savanna	North, South, East	Forest	Roots		Martins & Catarino 1258
*Anthocleista djalonensis* A.Chev.	Gentianaceae	Tree	Woodland, Savanna Woodland, Palm Grove	North, South, East, Bijagós	Sudanian/Guinean Sub-Region	Bark		Catarino 1717
*Anthocleista nobilis* G.Don	Gentianaceae	Tree	Woodland, Palm Grove, Gallery Forest	North, South, Bijagós	Afrotropical	Sap, Bark		Diniz et al. 1014
*Anthostema senegalense* A.Juss.	Euphorbiaceae	Tree	Forest, Woodland, Plam Grove, Gallery Forest	North, South, East, Bijagós	Sudanian/Guinean Sub-Region	Sap, Leaves	Phytochemical	Catarino 1553
*Argemone mexicana* L.	Papaveraceae	Herb	Ruderal	North, South, Bijagós	Introduced, Native to America	Leaves		Raimundo & Guerra 913
*Artabotrys velutinus* Scott-Elliot	Annonaceae	Shrub	Woodland, Savanna Woodland	North, South, East, Bijagós	Sudanian/Guinean Sub-Region	Leaves		Catarino 1085
*Asystasia gangetica* (L.) T.Anderson	Acanthaceae	Herb	Woodland, Savanna Woodland, Ruderal	North, South, East, Bijagós	Paleotropical	Flowers, Stem		Diniz et al. 2337
*Azadirachta indica* A.Juss.	Meliaceae	Tree	Cultivated	North	Introduced, Native To India	Leaves		Bancessi 17
*Bauhinia thonningii* Schum. (Syn. *Piliostigma thonningii* (Schumach. & Thonn.) Milne-Readh.)	Fabaceae	Tree	Woodland, Savanna Woodland, Palm Grove	North, South, East, Bijagós	Afrotropical	Roots, Bark	Handicrafts	Indjai 21
*Blighia sapida* K.D.Koenig	Sapindaceae	Tree	Woodland, Savanna Woodland	North, Bijagós	Guineo-Congolian Region	Leaves	Food	Diniz & Gonçalves 1946
*Blighia unijugata* Baker	Sapindaceae	Tree	Forest, Woodland, Gallery Forest	South	Afrotropical	Leaves	Food, Handicrafts	Diniz & Gonçalves 1845
*Blutaparon vermiculare* (L.) Mears	Amaranthaceae	Herb	Mangrove, Ruderal	North, South, East, Bijagós	Amphi-Atlantic	Whole plant		Catarino 650
*Borassus aethiopum* Mart.	Arecaceae	Palm	Woodland, Savanna Woodland	North, South, Bijagós	Afrotropical	Seeds, Roots	Food, Timber, Handicrafts	Pereira 1058
*Bridelia micrantha* (Hochst.) Baill.	Phyllanthaceae	Tree	Woodland, Savanna Woodland, Palm Grove, Gallery Forest	North, South, East, Bijagós	Afrotropical	Roots, Leaves	Food, Phytochemical	Indjai 20
*Caesalpinia benthamiana* (Baill.) Herend. & Zarucchi (Syn. *Mezoneuron benthamianum* Baill.)	Fabaceae	Climber	Forest, Woodland, Savanna Woodland, Palm Grove	North, South, East, Bijagós	Guineo-Congolian Region	Sap, Roots, Leaves		Catarino 1526
*Calotropis procera* (Aiton) Dryand.	Apocynaceae	Shrub	Savanna Woodland, Ruderal	North, South, East	Paleotropical	Roots		Diniz et al. 658
*Calycobolus heudelotii* (Baker ex Oliv.) Heine	Convolvulaceae	Climber	Forest, Woodland, Palm Grove	North, South, East	Guineo-Congolian Region	Stem, Leaves		Catarino 1713
*Calyptrochilum christyanum* (Rchb. f.) Summerh.	Orchidaceae	Herb	Woodland, Savanna Woodland, Palm Grove, Gallery Forest	North, South, East, Bijagós	Afrotropical	Whole plant		Indjai 35
*Capparis erythrocarpos* Isert	Capparaceae	Shrub	Woodland, Savanna Woodland, Gallery Forest	North, South, East, Bijagós	Afrotropical	Roots		Indjai 12
*Capsicum annuum* L. (Syn. *C. frutescens* L.)	Solanaceae	Herb	Cultivated	North, South, East, Bijagós	Introduced, Native to America	Aerial parts of plant	Food	Moreira 62
*Carapa procera* DC.	Meliaceae	Tree	Woodland, Savanna Woodland, Palm Grove, Gallery Forest	North, South, East, Bijagós	Afro-Neotropical	Seeds		Catarino 1566
*Carica papaya* L.	Caricaceae	Tree	Cultivated	North, South, East, Bijagós	Introduced, Native to America	Leaves, Roots	Food, Phytochemical	Moreira 22
*Cassia sieberiana* DC.	Fabaceae	Tree	Woodland, Savanna Woodland, Palm Grove	North, South, East, Bijagós	Sudanian Region	Rz, Fo		Catarino 818
*Cassytha filiformis* L.	Lauraceae	Herb	Woodland, Savanna Woodland, Palm Grove	North, South, Bijagós	Pantropical	Whole plant		Indjai 10
*Cayratia gracilis* (Guill. & Perr.) Suess.	Vitaceae	Herb	Forest, Woodland, Palm Grove	North, South, East, Bijagós	Afrotropical	Sap	Phytochemical	Vidigal et al. 151
*Ceiba pentandra* (L.) Gaertn.	Malvaceae	Tree	Forest, Woodland, Savanna Woodland	North, South, Bijagós	Pantropical	Bark	Timber	Catarino 1710
*Celosia argentea* L.	Amaranthaceae	Herb	Ruderal	South, East	Afrotropical	Branches	Food	Vidigal et al. 47
*Chamaecrista absus* (L.) H.S.Irwin & Barneby	Fabaceae	Herb	Woodland, Savanna Woodland, Wet Grass Savanna, Ruderal	North, South, East, Bijagós	Paleotropical	Whole plant		Diniz & Pinto-Basto 2404
*Chamaecrista nigricans* (Vahl) Greene	Fabaceae	Herb	Savanna Woodland	North, East	Paleotropical	Whole plant	Phytochemical	Catarino 1215
*Chrozophora senegalensis* (Lam.) A.Juss. ex Spreng.	Euphorbiaceae	Herb	Savanna Woodland	North	Sudanian/Guinean Sub-Region	Whole plant, Fruits		Catarino 928
*Cissampelos mucronata* A.Rich.	Menispermaceae	Climber	Woodland, Savanna Woodland, Palm Grove, Wet Grass Savanna	North, South, East, Bijagós	Afrotropical	Roots, Leaves, Stem		Indjai 11
*Cissus aralioides* (Welw. ex Baker) Planch.	Vitaceae	Climber	Forest, Woodland, Savanna Woodland, Wet Grass Savanna	North, South, East, Bijagós	Afrotropical	Leaves		Catarino 1413
*Cissus rufescens* Guill. & Perr.	Vitaceae	Climber	Woodland, Savanna Woodland, Wet Grass Savanna	South, East, Bijagós	Sudanian/Guineo-Congolian Regions	Leaves		Catarino 514
*Citrus limon* (L.) Osbeck	Rutaceae	Tree	Cultivated	North, South, East, Bijagós	Introduced, Native to Asia	Roots, Leaves	Food	Moreira 67
*Clerodendrum splendens* G.Don	Lamiaceae	Climber	Forest, Woodland, Savanna Woodland, Palm Grove	North, South, Bijagós	Guineo-Congolian Region	Roots		Catarino 1483
*Clerodendrum umbellatum* Poir.	Lamiaceae	Climber	Forest, Woodland, Savanna Woodland	South	Guineo-Congolian/Sudano-Zambezian Regions	Roots, Leaves		Diniz et al. 2572
*Cnestis ferruginea* Vahl ex DC.	Connaraceae	Shrub	Forest, Woodland, Palm Grove	North, South, East, Bijagós	Guineo-Congolian Region	Roots, Leaves, Fruits, Branches	Phytochemical	Indjai 24
*Cochlospermum tinctorium* Perrier ex A.Rich.	Bixaceae	Shrub	Woodland, Savanna Woodland	North, South, East	Sudanian/Guinean Sub-Region	Roots	Phytochemical	Catarino 1180
*Cola cordifolia* (Cav.) R.Br.	Malvaceae	Tree	Woodland, Savanna Woodland, Galery Forest	North, South, East	Sudanian Region	Seeds, Stem	Food, Handicrafts	Catarino 1431
*Cola nitida* (Vent.) Schott & Endl.	Malvaceae	Tree	Cultivated	North, South, East, Bijagós	Guinean Sub-Region	Seeds	Food	Moreira 72
*Combretum adenogonium* Steud. ex A.Rich.	Combretaceae	Shrub	Woodland, Savanna Woodland	North, South, East	Afrotropical	Bark		Catarino 1737
*Combretum collinum* Fresen.	Combretaceae	Shrub	Woodland, Savanna Woodland	North, South, East	Afrotropical	Roots		Catarino 1453
*Combretum lecardii* Engl. & Diels	Combretaceae	Shrub	Woodland, Savanna Woodland	North, East	Guinean Sub-Region	Leaves		Catarino 582
*Combretum micranthum* G.Don	Combretaceae	Shrub	Woodland, Savanna Woodland, Gallery Forest, Palm Grove	North, South, East, Bijagós	Sudanian/Guinean Sub-Region	Roots, Leaves	Beverages	Indjai 08
*Combretum nigricans* Lepr. ex Guill. & Perr.	Combretaceae	Tree	Woodland, Savanna Woodland	North, South, East, Bijagós	Sudanian Region	Leaves		Catarino 1508
*Conocarpus erectus* L.	Combretaceae	Shrub	Mangrove	North, South, Bijagós	Afro-Neotropical	Leaves		Catarino 326
*Cordyla pinnata* (Lepr. ex A.Rich.) Milne-Redh.	Fabaceae	Tree	Woodland, Savanna Woodland	North, South, East	Sudanian Region	Bark	Food	Catarino 1741
*Craterispermum laurinum* (Poir.) Benth.	Rubiaceae	Shrub	Forest, Palm Grove, Gallery Forest	North, South	Afrotropical	Bark		Catarino 1719
*Crossopteryx febrifuga* (Afzel. ex G.Don) Benth.	Rubiaceae	Shrub	Woodland, Savanna Woodland	North, South, East	Afrotropical	Leaves		Catarino 1725
*Cryptolepis sanguinolenta* (Lindl.) Schltr.	Apocynaceae	Climber	Woodland, Gallery Forest	North, South, Bijagós	Guineo-Congolian Region	Roots		Diniz et al. 995
*Curculigo pilosa* (Schumach. & Thonn.) Engl.	Hypoxidaceae	Herb	Woodland	North, South, East	Afrotropical	Roots		Pinto-Basto et al. 103
*Cymbopogon citratus* (DC.) Stapf	Poaceae	Herb	Cultivated, Ruderal	North	Introduced, Native to India	Aerial parts of plant		Moreira 216
*Dalbergia boehmii* Taub.	Fabaceae	Tree	Woodland, Savanna Woodland, Palm Grove	North, South, East, Bijagós	Sudanian/Zambezian Regions	Leaves		Catarino 1452
*Dalbergia saxatilis* Hook. f.	Fabaceae	Shrub	Woodland, Savanna Woodland, Gallery Forest	North, South, Bijagós	Guineo-Congolian Region	Leaves		Catarino 1636
*Daniellia oliveri* (Rolfe) Hutch. & Dalziel	Fabaceae	Tree	Savanna Woodland	North, South, East, Bijagós	Sudanian/Guineo-Congolian Regions	Leaves	Handicrafts, Phytochemical	Gomes & Correia 2
*Desmodium velutinum* (Willd.) DC.	Fabaceae	Shrub	Woodland, Savanna Woodland, Gallery Forest, Palm Grove	North, South, East, Bijagós	Paleotropical	Leaves, Roots		Catarino 1165
*Detarium microcarpum* Guill. & Perr.	Fabaceae	Tree	Woodland, Savanna Woodland, Palm Grove	North, South, East, Bijagós	Sudanian Region	Bark		Catarino 1724
*Dialium guineense* Willd.	Fabaceae	Tree	Forest, Woodland, Palm Grove, Gallery Forest	North, South, East, Bijagós	Sudanian/Guinean Sub-Region	Fruits, Leaves, Bark	Food	Diniz et al. 2531
*Dichrostachys cinerea* subsp*. platycarpa* (Welw. ex W.Bull) Brenan & Brummitt	Fabaceae	Shrub	Woodland, Savanna Woodland, Ruderal	North, South, East, Bijagós	Sudanian/Zambezian Regions	Bark		Catarino 1620
*Dicranolepis disticha* Planch.	Thymelaeaceae	Shrub	Forest, Woodland	South	Guineo-Congolian Region	Roots		Moreira 97
*Dioscorea hirtiflora* Benth.	Dioscoreaceae	Climber	Woodland, Savanna Woodland, Palm Grove	North, South, East, Bijagós	Afrotropical	Roots	Food	Catarino & Djalo 250
*Diospyros heudelotii* Hiern	Ebenaceae	Shrub	Forest, Woodland, Gallery Forest	North, South, East, Bijagós	Guinean Sub-Region	Bark		Catarino 1734
*Elaeis guineensis* Jacq.	Arecaceae	Palm	Forest, Woodland, Palm Grove, Gallery Forest	North, South, East, Bijagós	Sudanian/Guineo-Congolian Regions	Roots	Food, Timber, Handicrafts	Diniz et al. 1031
*Eleocharis mutata* (L.) Roem. & Schult.	Cyperaceae	Herb	Gallery Forest, Wet Grass Savanna	North, South, East, Bijagós	Amphi-Atlantic	Ca, Seeds		Vidigal et al. 96
*Entada africana* Guill. & Perr.	Fabaceae	Shrub	Savanna Woodland	South, East	Sudanian/Zambezian Regions	Roots, Bark		Catarino 1378
*Erythrina senegalensis* DC.	Fabaceae	Tree	Woodland, Savanna Woodland, Palm Grove	North, South, East, Bijagós	Sudanian Region	Bark, Roots	Handicrafts	Indjai 04
*Erythrina sigmoidea* Hua	Fabaceae	Tree	Savanna Woodland	East	Sudanian Region	Roots		Catarino 1389
*Faidherbia albida* (Delile) A. Chev.	Fabaceae	Tree	Woodland, Savanna Woodland, Palm Grove	North, East, Bijagós	Paleotropical	Bark		Indjai 23
*Ficus exasperata* Vahl	Moraceae	Tree	Forest, Woodland, Palm Grove, Gallery Forest	North, South, Bijagós	Paleotropical	Bark, Leaves		Indjai 14
*Ficus lutea* Vahl	Moraceae	Tree	Forest, Woodland, Palm Grove	North, South, Bijagós	Afrotropical	–	Phytochemical, Rituals and beliefs	Catarino 1654
*Ficus polita* Vahl	Moraceae	Tree	Forest, Palm Grove	North, South, Bijagós	Afrotropical	Leaves, Bark		Indjai 01
*Ficus sur* Forssk.	Moraceae	Tree	Forest, Woodland, Savanna Woodland, Palm Grove, Gallery Forest	North, South, East, Bijagós	Afrotropical	Roots, Bark, Fruits	Food	Catarino 1574
*Fleroya stipulosa* (DC.) Y.F.Deng (Syn. *Hallea stipulosa* (DC.) Leroy)	Rubiaceae	Tree	Gallery Forest	North, Bijagós	Guineo-Congolian Region	Bark		Espírito Santo 426
*Funtumia africana* (Benth.) Stapf	Apocynaceae	Tree	Woodland, Savanna Woodland, Gallery Forest	North, South	Afrotropical	Roots		Diniz et al. 2557
*Gardenia ternifolia* subsp. *jovis-tonantis* (Welw.) Verdc.	Rubiaceae	Shrub	Woodland, Savanna Woodland	North, South, East	Sudanian/Zambezian Regions	Roots		Catarino 1373
*Guiera senegalensis* J.F.Gmel.	Combretaceae	Shrub	Savanna Woodland, Ruderal	North, South, East, Bijagós	Sahelian/Sudanian Regions	Leaves, Roots		Catarino 1926
*Gymnanthemum coloratum* (Willd.) H.Rob. & B.Kahn (Syn. *Vernonia colorata* (Willd.) Drake)	Asteraceae	Shrub	Woodland	North, South, East, Bijagós	Afrotropical	Roots, Leaves		Diniz & Gonçalves 1847
*Gymnema sylvestre* (Retz.) R.Br. ex Sm.	Apocynaceae	Climber	Woodland, Gallery Forest	North, South, East, Bijagós	Afrotropical	Bark		Catarino 1347
*Gymnosporia senegalensis* (Lam.) Loes. (Syn. *Maytenus senegalensis* (Lam.) Exell)	Celastraceae	Shrub	Savanna Woodland, Gallery Forest, Wet Grass Savanna	North, East	Sudanian/Zambezian Regions	Leaves		Saneé 19
*Harungana madagascariensis* Lam. ex Poir.	Hypericaceae	Shrub	Woodland, Savanna Woodland, Palm Grove, Gallery Forest	North, South, East, Bijagós	Afrotropical	Leaves		Catarino 1542
*Hibiscus sterculiifolius* (Guill. & Perr.) Steud.	Malvaceae	Herb	Woodland, Savanna Woodland, Palm Grove, Gallery Forest, Ruderal	North, South, East, Bijagós	Sudanian/Guinean Sub-Region	Leaves	Handicrafts	Catarino 1649
*Holarrhena floribunda* (G.Don) T. Durand & Schinz	Apocynaceae	Tree	Woodland, Savanna Woodland, Gallery Forest, Palm Grove	North, South, East, Bijagós	Guineo-Congolian Region	Leaves, Bark	Handicrafts	Catarino 1706
*Hymenocardia acida* Tul.	Phyllanthaceae	Tree	Woodland, Savanna Woodland	North, South, East, Bijagós	Afrotropical	Bark, Leaves		Indjai 32
*Hypoestes forsskaolii* (Vahl) R.Br.	Acanthaceae	Herb	Savanna Woodland, Ruderal	North	Afrotropical	Roots		Diniz & Gonçalves 1820
*Hyptis spicigera* Lam.	Lamiaceae	Herb	Savanna Woodland, Gallery Forest, Palm Grove, Ruderal	North, South, East	Introduced, Native to America	Leaves		Catarino 1193
*Hyptis suaveolens* (L.) Poit.	Lamiaceae	Herb	Woodland, Savanna Woodland, Gallery Forest, Palm Grove, Ruderal	North, South, East, Bijagós	Introduced, Native to America	Aerial parts of plant	Phytochemical	Diniz & Catarino 1698
*Icacina oliviformis* (Poir.) J.Raynal	Icacinaceae	Shrub	Savanna Woodland	North, South, East, Bijagós	Sudanian/Guinean Sub-Region	Roots	Food	Diniz & Gonçalves 1923
*Indigofera arrecta* Hochst. ex A.Rich.	Fabaceae	Herb	Cultivated, Ruderal	North, East,	Paleotropical	Aerial parts of plant	Phytochemical	Espírito Santo 2304
*Indigofera macrophylla* Schumach. & Thonn.	Fabaceae	Herb	Woodland, Palm Grove, Gallery Forest	North, South, East, Bijagós	Sudanian/Guinean Sub-Region	Roots		Catarino 824
*Indigofera suffruticosa* Mill.	Fabaceae	Shrub	Cultivated	North	Introduced, Native to America	Aerial parts of plant	Phytochemical	Espírito Santo 2278
*Ipomoea asarifolia* (Desr.) Roem. & Schult.	Convolvulaceae	Herb	Woodland, Palm Grove	North, South, Bijagós	Pantropical	Leaves		Catarino 770
*Jatropha curcas* L.	Euphorbiaceae	Shrub	Cultivated	South, Bijagós	Introduced, Native to America	Roots, Seeds		Vidigal et al. 191
*Jatropha gossypiifolia* L.	Euphorbiaceae	Shrub	Cultivated	South	Introduced, Native to America	Sap		Moreira 206
*Kalanchoe crenata* (Andrews) Haw.	Crassulaceae	Herb	Ruderal	South	Paleotropical	Sap		Moreira 242
*Khaya senegalensis* (Desv.) A.Juss.	Meliaceae	Tree	Woodland, Savanna Woodland, Ruderal	North, South, East, Bijagós	Sudanian/Guinean Sub-Region	Bark	Timber	Indjai 22
*Landolphia dulcis* (R.Br. ex Sabine) Pichon	Apocynaceae	Climber	Woodland, Savanna Woodland, Gallery Forest, Palm Grove	North, South, East, Bijagós	Guineo-Congolian Region	Roots	Food, Phytochemical	Indjai 25
*Landolphia heudelotii* A.DC.	Apocynaceae	Climber	Woodland, Savanna Woodland, Gallery Forest, Palm Grove	North, South, East, Bijagós	Sudanian/Guinean Sub-Region	Leaves, Bark, Branches	Food, Phytochemical	Catarino 1594
*Lannea acida* A.Rich.	Anacardiaceae	Tree	Savanna Woodland	North, South, East, Bijagós	Sudanian Region	Bark	Food, Phytochemical	Catarino 2130
*Lecaniodiscus cupanioides* Planch. ex Benth.	Sapindaceae	Shrub	Forest, Woodland, Palm Grove, Gallery Forest	North, South	Guineo-Congolian Region	Roots	Food	Catarino 1665
*Leonotis nepetifolia* (L.) R.Br.	Lamiaceae	Herb	Savanna Woodland, Ruderal	North, Bijagós	Afrotropical	Aerial parts of plant		Diniz & Gonçalves 1872
*Lepisanthes senegalensis* (Poir.) Leenh. (Syn. *Aphania senegalensis* (A.Juss. ex Poir.) Radlk.)	Sapindaceae	Shrub	Woodland, Savanna Woodland, Palm Grove	North, East, Bijagós	Guineo-Congolian Region	Leaves	Food	Catarino 1715
*Leptadenia lancifolia* (Schumach. & Thonn.) Decne. (Syn. *L. hastata* (Pers.) Vatke)	Apocynaceae	Climber	Woodland, Savanna Woodland	North, South, East	Sudanian/Guinean Sub-Region	Stem, Branches, Leaves		Indjai 34
*Leptoderris brachyptera* (Benth.) Dunn	Fabaceae	Climber	Forest, Woodland, Savanna Woodland, Gallery Forest	North, South, East, Bijagós	Guineo-Congolian Region	Leaves		Catarino 1109
*Lippia chevalieri* Moldenke	Verbenaceae	Shrub	Woodland, Savanna Woodland	North, South, East	Sudanian Region	Roots		Catarino 1387
*Lonchocarpus sericeus* (Poir.) KunthDC.	Fabaceae	Tree	Gallery Forest, Mangrove	North, South, East, Bijagós	Afro-Neotropical	Bark		Catarino 740
*Luffa cylindrica* (L.) M.Roem.	Cucurbitaceae	Herb	Cultivated, Also Ruderal	North, East, Bijagós	Introduced, Native to Asia	Leaves		Catarino 1367
*Maerua duchesnei* (De Wild.) F.White	Capparaceae	Shrub	Forest, Woodland, Gallery Forest	South	Sudanian/Guineo-Congolian Regions	Leaves		Catarino 1588
*Mangifera indica* L.	Anacardiaceae	Tree	Cultivated	North, South, East, Bijagós	Introduced, Native to Asia	Roots, Leaves, Bark	Food	Diniz & Gonçalves 1939
*Margaritaria discoidea* (Baill.) G.L.Webster	Phyllanthaceae	Tree	Forest, Woodland, Savanna Woodland, Palm Grove, Gallery Forest	North, South, Bijagós	Afrotropical	Leaves, Bark		Indjai 27
*Mitragyna innermis* (Willd.) Kuntze	Rubiaceae	Tree	Gallery Forest, Wet Grass Savanna	North, South, East	Sudanian/Guineo-Congolian Regions	Leaves		Catarino 1426
*Momordica cissoides* Planch. ex Benth.	Cucurbitaceae	Herb	Forest, Gallery Forest	North, South, East	Afrotropical	Sap		Moreira 61
*Monodora myristica* (Gaertn.) Dunal	Annonaceae	Tree	Forest, Gallery Forest	North, South, East, Bijagós	Afrotropical	Seeds		Espírito Santo 2331
*Morinda chrysorhiza* (Thonn.) DC. (Syn. *M. geminata* DC.)	Rubiaceae	Tree	Woodland, Savanna Woodland, Palm Grove	North, South, East, Bijagós	Guineo-Congolian Region	Leaves, Branches	Phytochemical	Catarino 1534
*Moringa oleifera* Lam.	Moringaceae	Tree	Cultivated	North, South, East, Bijagós	Introduced, Native to India	Leaves	Food	Vidigal et al. 198
*Neocarya macrophylla* (Sabine) Prance ex F.White	Chrysobalanaceae	Tree	Woodland, Savanna Woodland, Palm Grove	North, South, East, Bijagós	Sudanian Region	Leaves, Fruits, Bark	Food	Diniz & Gonçalves 1778
*Newbouldia laevis* (P.Beauv.) Seem.	Bignoniaceae	Tree	Woodland, Savanna Woodland, Palm Grove	North, South, East, Bijagós	Guineo-Congolian Region	Roots, Leaves		Indjai 03
*Ocimum basilicum* L.	Lamiaceae	Herb	Savanna Woodland, Ruderal	North, East, Bijagós	Pantropical	Aerial parts of plant	Phytochemical	Indjai 36
*Ocimum gratissimum* L.	Lamiaceae	Shrub	Palm Grove, Ruderal	North, South, Bijagós	Introduced, Native to Asia	Aerial parts of plant	Beverages	Indjai 37
*Opilia amentacea* Roxb.	Opiliaceae	Climber	Savanna Woodland	South, East	Afrotropical	Roots, Leaves	Rituals and beliefs	Espirito Santo 2352
*Ozoroa insignis* Delile	Anacardiaceae	Tree	Savanna Woodland	North, South, East, Bijagós	Afrotropical	Roots		Catarino 513
*Parinari excelsa* Sabine	Chrysobalanaceae	Tree	Forest, Woodland, Palm Grove	North, South, East, Bijagós	Afro-Neotropical	Bark	Food, Timber	Catarino 1705
*Parkia biglobosa* (Jacq.) G.Don	Fabaceae	Tree	Woodland, Savanna Woodland	North, South, East, Bijagós	Afro-Neotropical	Bark	Food	Diniz & Gonçalves 1790
*Paulinia pinnata* L.	Sapindaceae	Climber	Forest, Woodland, Savanna Woodland, Palm Grove, Gallery Forest	North, South, East, Bijagós	Afro-Neotropical	Stem, Leaves		Catarino 503
*Pavetta corymbosa* (DC.) F.N.Williams	Rubiaceae	Shrub	Forest, Woodland, Palm Grove, Gallery Forest	North, South, Bijagós	Sudanian/Guinean Sub-Region	Leaves		Catarino 1619
*Pavetta oblongifolia* (Hiern) Bremek.	Rubiaceae	Shrub	Woodland, Savanna Woodland	North, South, East	Sudanian Region	Branches, Leaves		Catarino 1437
*Pericopsis laxiflora* (Benth. ex Baker) Meeuwen	Fabaceae	Tree	Woodland, Savanna Woodland	North, South, East	Sudanian Region	Bark	Handicrafts	Catarino 1736A
*Phoenix reclinata* Jacq.	Arecaceae	Palm	Gallery Forest, Mangrove	North, South, East, Bijagós	Afrotropical	Roots	Food, Handicrafts	Martins & Catarino 1404
*Phyllanthus muellerianus* (Kuntze) Exell	Phyllanthaceae	Shrub	Forest, Woodland, Savanna Woodland, Palm Grove, Gallery Forest	North, South, Bijagós	Afrotropical	Leaves		Catarino 1615
*Piper guineense* Schumach. & Thonn.	Piperaceae	Climber	Forest, Woodland, Gallery Forest	South, East, Bijagós	Afrotropical	Fruits		Diniz & Gonçalves 2083
*Platostoma africanum* P.Beauv.	Lamiaceae	Herb	Savanna Woodland, Ruderal	South, East	Paleotropical	Aerial parts of plant	Food	Gonçalves et al. 90
*Pouchetia africana* A.Rich. ex DC.	Rubiaceae	Tree	Forest, Woodland, Palm Grove	North, South, Bijagós	Sudanian/Guinean Sub-Region	Leaves		Catarino 1632
*Pouteria alnifolia* (Baker) Roberty (Syn. *Malacantha alnifolia* (Baker) Pierre)	Sapotaceae	Tree	Forest, Woodland, Savanna Woodland, Palm Grove	North, South, East, Bijagós	Afrotropical	Roots		Catarino 1721
*Premna hispida* Benth.	Lamiaceae	Shrub	Forest, Woodland, Wet Grass Savanna	North, South	Sudanian/Guinean Sub-Region	Leaves		Catarino 1100
*Prosopis africana* (Guill. & Perr.) Taub.	Fabaceae	Tree	Woodland, Savanna Woodland, Palm Grove	North, South, East, Bijagós	Sudanian/Guineo-Congolian Regions	Leaves, Bark, Roots	Timber	Catarino 1070
*Psorospermum corymbiferum* Hochr.	Hypericaceae	Shrub	Woodland	South, East	Guineo-Congolian/Sudano-Zambezian Regions	Leaves		Espirito Santo 533
*Psorospermum glaberrimum* Hochr.	Hypericaceae	Shrub	Woodland, Savanna Woodland	South, East	Guinean Sub-Region	Leaves	Beverages	Moreira 124
*Psychotria peduncularis* (Salisb.) Steyerm.	Rubiaceae	Shrub	Forest, Woodland, Savanna Woodland, Palm Grove, Gallery Forest	North, South, Bijagós	Afrotropical	Leaves, Roots		Indjai 07
*Psydrax parviflora* (Afzel.) Bridson	Rubiaceae	Shrub	Woodland, Gallery Forest	South	Afrotropical	Leaves, Bark		Martins & Catarino 1181
*Pterocarpus erinaceus* Lam. ex Poir.	Fabaceae	Tree	Woodland, Savanna Woodland	North, South, East	Sudanian Region	Bark	Timber	Catarino 1691
*Pterocarpus santalinoides* L׳Hér. ex DC.	Fabaceae	Tree	Gallery Forest	North, South, East, Bijagós	Afro-Neotropical	Roots	Food	Catarino 1692
*Quassia undulata* (Guill. & Perr.) D.Dietr. (Syn. *Hannoa undulata* (Guill. & Perr.) Planch.)	Simaroubaceae	Tree	Woodland, Savanna Woodland	North, South, East, Bijagós	Sudanian/Guinean Sub-Regions	Leaves	Rituals and beliefs	Catarino 1440
*Rauvolfia vomitoria* Afzel.	Apocynaceae	Tree	Forest, Woodland, Savanna Woodland, Gallery Forest	North, South, East, Bijagós	Afrotropical	Leaves, Roots, Stem		Catarino 1690
*Ricinus communis* L.	Euphorbiaceae	Herb	Cultivated	North, East, Bijagós	Paleotropical	Seeds		Diniz & Gonçalves 1915A
*Ritchiea capparoides* (Andrews) Britten	Capparaceae	Shrub	Forest, Woodland, Savanna Woodland, Palm Grove	North, South, Bijagós	Afrotropical	Roots		Catarino 601
*Saba senegalensis* (A.DC.) Pichon	Apocynaceae	Climber	Forest, Woodland, Savanna Woodland, Palm Grove	North, South, East, Bijagós	Sudanian/Guinean Sub-Region	Leaves	Food, Rituals and beliefs	Catarino 1577
*Salacia senegalensis* (Lam.) DC.	Celastraceae	Climber	Forest, Woodland, Palm Grove	North, South, East, Bijagós	Guineo-Congolian Region	Roots	Food	Catarino 1650
*Samanea dinklagei* (Harms) Keay (Syn. *Albizia dinklagei* (Harms) Harms)	Fabaceae	Tree	Woodland, Savanna Woodland, Gallery Forest, Palm Grove, Wet Grass Savanna	North, South, East, Bijagós	Guineo-Congolian Region	Bark	Handicrafts	Diniz et al. 1268
*Sansevieria senegambica* Baker	Asparagaceae	Herb	Forest, Woodland, Palm Grove	North, South, Bijagós	Guinean Sub-Region	Roots	Handicrafts, Phytochemical	Catarino 692
*Sarcocephalus latifolius* (Sm.) E.A.Bruce	Rubiaceae	Shrub	Forest, Woodland, Palm Grove, Gallery Forest, Wet Grass Savanna	North, South, East, Bijagós	Sudanian/Guineo-Congolian Regions	Roots, Bark, Leaves	Food	Catarino 1344
*Schultesia guianensis* var*. latifólia* (Mart. ex Progel) E.F.Guim. & Fontella (Syn. *S. stenophylla* var. *latifolia* Mart. ex Progel)	Gentianaceae	Herb	Wet Grass Savanna, Ruderal	North, South, East	Guinean Sub-Region	Whole plant		Diniz & Catarino 1674
*Scleria boivinii* Steud.	Cyperaceae	Herb	Woodland, Savanna Woodland	North, South	Afrotropical	Whole plant		Diiz & Gonçalves 1792
*Sclerocarya birrea* (A.Rich.) Hochst.	Anacardiaceae	Shrub	Savanna Woodland	North, South, East, Bijagós	Afrotropical	Bark, Leaves		Gonçalves et al. 83
*Scoparia dulcis* L.	Plantaginaceae	Herb	Woodland, Palm Grove, Wet Grass Savanna, Ruderal	North, South, East, Bijagós	Pantropical	Leaves, Stem		Catarino 696
*Secamone afzelii* (Roem. & Schult.) K.Schum.	Apocynaceae	Climber	Woodland, Savanna Woodland, Palm Grove	North, South, Bijagós	Guineo-Congolian Region	Roots, Leaves		Catarino 1084
*Securidaca longipedunculata* Fresen.	Polygalaceae	Shrub	Woodland, Savanna Woodland	North, South, East, Bijagós	Afrotropical	Leaves, Roots	Rituals and beliefs	Catarino 1350
*Senna alata* (L.) Roxb.	Fabaceae	Shrub	Ruderal	North, South, East	Introduced, Native to America	Leaves	Beverages	Diniz & Catarino 1728
*Senna obtusifolia* (L.) H.S. Irwin & Barneby	Fabaceae	Shrub	Ruderal	North, South, East, Bijagós	Introduced, Native to America	Leaves		Vidigal et al. 131
*Senna occidentalis* (L.) Link	Fabaceae	Herb	Ruderal	North, South, East, Bijagós	Pantropical	Leaves, Roots	Beverages	Diniz et al. 996
*Senna podocarpa* (Guill. & Perr.) Lock	Fabaceae	Shrub	Woodland, Gallery Forest, Palm Grove,	North, South, East, Bijagós	Guineo-Congolian Region	Leaves		Indjai 02
*Senna siamea* (Lam.) H.S. Irwin & Barneby	Fabaceae	Tree	Savanna Woodland, Cultivated	North, South	Introduced, Native to Asia	Leaves		Moreira 26
*Sida linifolia* Juss. ex Cav.	Malvaceae	Herb	Woodland, Savanna Woodland, Wet Grass Savanna, Ruderal	North, South, East, Bijagós	Afro-Neotropical	Aerial parts of plant		Catarino 1054
*Sida urens* L.	Malvaceae	Herb	Gallery Forest, Ruderal	North, South, East	Pantropical	Aerial parts of plant		Catarino 1403
*Smeathmannia laevigata* Sol. ex R.Br.	Passifloraceae	Shrub	Woodland, Palm Grove, Gallery Forest	North, South, East, Bijagós	Sudanian/Guinean Sub-Region	Branches		Catarino 1651
*Smilax anceps* Willd.	Smilacaceae.	Climber	Woodland, Savanna Woodland, Palm Grove	North, South, East, Bijagós	Afrotropical	Leaves, Roots		Catarino 1380
*Solanum macrocarpon* L.	Solanaceae	Herb	Cultivated	North, South, East, Bijagós	Afro-Neotropical	Roots, Leaves	Food	Diniz et al. 1054
*Sorindeia juglandifolia* (A.Rich.) Planch. ex Oliv.	Anacardiaceae	Shrub	Woodland, Savanna Woodland	North, South, East, Bijagós	Afrotropical	Leaves	Food	Catarino 2129
*Spermacoce verticillata* L.	Rubiaceae	Herb	Savanna Woodland, Wet Grass Savanna, Ruderal	North, South, East, Bijagós	Afrotropical	Roots		Catarino 790
*Sphaeranthus senegalensis* DC.	Asteraceae	Herb	Palm Grove, Ruderal	North, South, East, Bijagós	Paleotropical	Whole plant		Catarino 1602
*Spondias mombin* L.	Anacardiaceae	Tree	Woodland, Savanna Woodland	North, South, East, Bijagós	Afro-Neotropical	Leaves	Food	Catarino 701
*Stereospermum kunthianum* Cham.	Bignoniaceae	Tree	Woodland, Savanna Woodland	North, South, East	Afrotropical	Bark		Martins & Catrino 1500
*Strophanthus hispidus* DC.	Apocynaceae	Climber	Woodland, Savanna Woodland, Gallery Forest, Palm Grove	North, South, East	Guineo-Congolian Regions	Roots		Catarino 734
*Strophanthus sarmentosus* DC.	Apocynaceae	Climber	Forest, Woodland, Savanna Woodland, Palm Grove	North, South, East, Bijagós	Sudanian/Guineo-Congolian Regions	Roots	Rituals and beliefs	Indjai 13
*Tapinanthus bangwensis* (Engl. & K.Krause) Danser	Loranthaceae	Shrub	Woodland, Savanna Woodland	North, South, East, Bijagós	Sudanian/Guineo-Congolian Regions	Whole plant	Rituals and Beliefs	Catarino 1461
*Terminalia albida* Scott-Elliot	Combretaceae	Tree	Woodland, Savanna Woodland	North, South, East, Bijagós	Sudanian/Guinean Sub-Region	Leaves		Catarino 1925
*Terminalia macroptera* Guill. & Perr.	Combretaceae	Tree	Woodland, Savanna Woodland	North, South, East, Bijagós	Sudanian Region	Leaves, Bark		Catarino 1923
*Tetracera alnifolia* Willd.	Dilleniaceae	Climber	Woodland, Savanna Woodland, Palm Grove	North, East, Bijagós	Afrotropical	Sap		Catarino 1104
*Trema orientalis* (L.) Blume	Cannabaceae	Tree	Forest, Woodland, Palm Grove	North, South, East, Bijagós	Paleotropical	Leaves		Catarino 1617
*Trichilia emetica* subsp. *suberosa* J.J.de Wilde	Meliaceae	Tree	Forest, Woodland, Savanna Woodland	North, South, East	Paleotropical	Bark		Catarino 622
*Trichilia prieuriana* A.Juss.	Meliaceae	Tree	Forest, Woodland, Savanna Woodland, Palm Grove, Gallery Forest	North, South, East, Bijagós	Guineo-Congolian Region	Leaves, Bark		Indjai 19
*Triclisia patens* Oliv.	Menispermaceae	Climber	Forest, Woodland, Palm Grove	North, South, East	Guinean Sub-Region	Roots	Food	Catarino 1565
*Triumfetta cordifolia* A.Rich.	Malvaceae	Shrub	Woodland, Savanna Woodland, Palm Grove, Wet Grass Savanna, Ruderal	North, South, East, Bijagós	Afrotropical	Leaves		Catarino & Bancessi 659
*Urena lobata* L.	Malvaceae	Herb	Woodland, Savanna Woodland, Palm Grove, Ruderal	North, South, East, Bijagós	Pantropical	Leaves	Handicrafts	Catarino 1407
*Usteria guineensis* Willd.	Loganiaceae	Climber	Forest, Woodland, Savanna Woodland, Palm Grove	North, South, East, Bijagós	Sudanian/Guineo-Congolian Regions	Leaves		Catarino 1672
*Uvaria chamae* P.Beauv.	Annonaceae	Climber	Woodland, Savanna Woodland	North, South, East, Bijagós	Sudanian/Guineo-Congolian Regions	Roots, Branches, Leaves	Food	Diniz & Gonçalves 1908
*Vernonia nigritana* Oliv. & Hiern	Asteraceae	Herb	Woodland, Savanna Woodland	North, South, East	Sudanian/Guinean Sub-Region	Roots		Catarino 1439
*Vigna unguiculata* subsp. *unguiculata* var. *spontanea* (Schweinf.) Pasquet	Fabaceae	Herb	Woodland, Savanna Woodland, Wet Grass Savanna	North, South, Bijagós	Afrotropical	Leaves	Food	Diniz & Pinto-Basto 2407
*Vitex doniana* Sweet	Lamiaceae	Tree	Woodland, Savanna Woodland, Gallery Forest	North, South, East, Bijagós	Sudanian/Guineo-Congolian Regions	Roots	Food	Catarino 1424
*Vitex madiensis* Oliv.	Lamiaceae	Tree	Woodland, Savanna Woodland	North, South, East	Guineo-Congolian/Sudano-Zambezian Regions	Roots, Flowers	Food	Catarino 1660
*Voacanga africana* Stapf ex Scott-Elliot	Apocynaceae	Tree	Forest, Woodland, Palm Grove	South, Bijagós	Afrotropical	Roots, Leaves, Bark		Catarino 1714
*Waltheria indica* L.	Malvaceae	Shrub	Woodland, Savanna Woodland, Palm Grove, Gallery Forest	North, South, East, Bijagós	Pantropical	Roots		Catarino 1065
*Ximenia americana* L.	Olacaceae	Tree	Forest, Woodland, Savanna Woodland, Palm Grove, Gallery Forest	North, South, East, Bijagós	Pantropical	Roots, Bark	Food, Phytochemical	Catarino 1605
*Xylopia aethiopica* (Dunal) A.Rich.	Annonaceae	Tree	Forest, Woodland	North, South, East, Bijagós	Afrotropical	Leaves, Fruits, Bark	Food	Catarino 879
*Xylopia longipetala* De Wild. & T.Durand	Annonaceae	Shrub	Woodland, Savanna Woodland, Gallery Forest	North, South, East, Bijagós	Afrotropical	Leaves	Food	Diniz & Gonçalves 1912
*Zanthoxylum leprieurii* Guill. & Perr.	Rutaceae	Tree	Forest, Woodland, Savanna Woodland, Palm Grove, Gallery Forest	North, South, East, Bijagós	Sudanian/Guineo-Congolian Regions	Bark, Leaves, Roots		Indjai 09
*Zanthoxylum zanthoxyloides* (Lam.) Zepern. & Timler	Rutaceae	Tree	Forest, Woodland, Savanna Woodland, Palm Grove, Gallery Forest	North, South, Bijagós	Sudanian/Guinean Sub-Region	Roots		Catarino 926
